# Physical activity and TV viewing parenting practices for toddlers among South Asian and white families in the UK: born in Bradford 1000 study

**DOI:** 10.1186/s12889-023-16522-w

**Published:** 2023-08-22

**Authors:** Soyang Kwon, Namratha R. Kandula, Pooja S. Tandon, Nilay S. Shah

**Affiliations:** 1 Department of Pediatrics, Northwestern University, 225 E Chicago Ave. Box 157, Chicago, IL 60611 USA; 2 Department of Medicine and Preventive Medicine, Northwestern University, Chicago, IL 60611 USA; 3 University of Washington & Seattle Children’s Hospital, M/S CURE-3, PO Box 5371, Seattle, WA 98145 USA; 4 Department of Medicine (Cardiology) and Preventive Medicine, Northwestern University, Chicago, IL 60611 USA

**Keywords:** Early childhood, Children, Mothers, Acculturation, Walking, Sedentary behavior, Pakistani immigrants

## Abstract

**Background:**

Children of South Asian (SA) origin in the UK have lower levels of physical activity (PA), compared to their White counterparts. Parents play an important role in establishing PA habits among young children. The aim of this study was to compare PA and television (TV) viewing parenting practices for young children between SA British (SAB) and White British (WB) parents living in the UK.

**Methods:**

We conducted a secondary analysis of the Born in Bradford (BiB) 1000 study, using survey data at child ages 24 and 36 months. The study sample included three groups of mothers (*n* = 1,149): foreign-born SAB (*n* = 458), UK-born SAB (*n* = 276), and WB (*n* = 455). Mothers completed a survey about parenting practices (i.e., PA supports, PA restrictions, TV viewing restrictions) at child age 24 months and child PA and TV viewing behaviors at child ages 24 and 36 months. Parenting practices were compared among the three groups. Multivariable linear regression analyses compared children’s weekly walking frequency and daily TV viewing hours by parenting practices in the three groups.

**Results:**

The foreign-born SAB group showed the lowest frequencies of PA-supportive parenting practices (verbal encouragement: 3.7 ± 3.1 times/week; logistic support: 1.5 ± 1.8 times/week) and the highest frequencies of PA-restrictive parenting practices (7.8 ± 7.7 times/week) among the three groups (*p* < 0.01). Children of Foreign-born SAB mothers had the most frequent TV watching during a mealtime (4.0 ± 3.1 times/week) among the three groups (*p* < 0.01). Less frequent PA-supportive parenting practices and SA ethnicity were associated with lower walking frequency at 24 and 36 months of age among children (*p* < 0.01). More frequent exposure to TV at mealtimes and SA ethnicity were associated with higher TV viewing time at 24 and 36 months of age among children (*p* < 0.01).

**Conclusions:**

This study demonstrated that SAB parents, particularly those who are foreign-born, apply parenting practices for their young children that are less supportive of PA and more supportive of TV viewing, and their children have lower PA and higher TV viewing time, compared with their WB counterparts.

**Supplementary Information:**

The online version contains supplementary material available at 10.1186/s12889-023-16522-w.

## Introduction

People of South Asian (SA) ethnicity are individuals who have ethnic, historical, or cultural origins in the countries of Bangladesh, Bhutan, India, Nepal, Pakistan, and Sri Lanka [1]. People of SA background have higher risks of cardiovascular disease, type 2 diabetes, and high blood pressure, compared to White and other Asian groups [2–5]. Physical activity (PA) has been recognized as a modifiable behavior to reduce cardiometabolic risks [6]. However, SA immigrants living in the United Kingdom (UK) participate in lower levels of PA, compared to their White counterparts [7]. Further, first-generation (born outside the UK) SA British (SAB) people are less physically active than the second-generation (born in the UK) [7]. Some SA immigrants describe unique religious and cultural barriers to PA engagement (e.g., inability to wear parts of their religious attire in conventional recreational spaces [1]; unfamiliarity and misconceptions about PA [8], prioritization of family and work over PA [8]; and beliefs regarding gender norms that PA is not appropriate for women or girls [9]).

Physical inactivity has also been highlighted among SAB children. While physical inactivity is a concern for both SAB and White British (WB) children, SAB children are even less active and more sedentary, compared to WB children [10–14]. PA and sedentary behaviors (e.g., television [TV] viewing) among SAB children are likely partly influenced by parents’ attitudes and beliefs [7], which are potentially transmitted via parenting practices [13]. Studies [7, 15] identified constraints on children’s time for PA, limited parental support for child PA, and PA having a low priority as barriers to PA in SAB school-aged and older children, which are similar to barriers experienced by families across demographic groups. A qualitative study [14] reported that SAB parents of school-aged children expressed that “we don’t really make plan to go out” and pointed that during extended family gatherings, TV or screen devices were used to keep children occupied and adults and children also watched TV together. Although physical inactivity and sedentary behavior habits are established in early childhood and tend to be sustained [16], these parenting practices among SAB parents of young children have not been evaluated.

To address the knowledge gap, this study aimed to evaluate PA and TV viewing parenting practices for young children among foreign-born SAB, UK-born SAB, and WB families. We hypothesized that SAB parents less frequently engage in parenting practices that encourage PA and limit TV viewing and more frequently engage in parenting practices that restrict PA and encourage TV viewing, compared with WB parents. An exploratory aim was to examine whether PA-related parenting practices differ by child sex among SAB parents. The secondary aim was to examine the associations of SA ethnicity and parenting practices with child PA and TV viewing behaviors. Knowledge about PA and TV viewing parenting practices specific to the SA population will help to identify culturally tailored and acceptable PA promotion strategies for SA families.

## Methods

We conducted a secondary data analysis using data from the Born in Bradford (BiB) 1000 study. The BiB study is a prospective birth cohort study that has longitudinally collected health and well-being information from the children and families of 12,453 women who were recruited at a gestational age of 26–28 weeks in Bradford, UK, between 2007 and 2010 [17]. Bradford is a city with high levels of socio-economic deprivation and ethnic diversity. The BiB 1000 study is a sub-study of the BiB study, which aimed to investigate growth trajectories and identify risk factors for childhood obesity in early childhood [17, 18]. The BiB 1000 study included 1,735 BiB mother participants who were recruited between August 2008 and March 2009 and conducted surveys when their children were 6, 12, 18, 24, and 36 months. Because mothers, than fathers, primarily support child PA [19], we used maternal ethnicity to define the family’s ethnic group. Maternal ethnicity was determined by self-identified ethnicity at baseline (gestational age of 26–28 weeks) [13]. Of the 1,735 BiB 1000 mother participants, 971 (56.0%) were SAB and 670 (38.6%) were WB (English, Walsh, Scottish, Northern Irish, or British). We further divided the SAB group into foreign-born and UK-born, based on the mother’s birthplace. For this secondary analysis, we included SAB or WB mothers who completed the 24-month survey and used their data obtained from the 24- and 36-month surveys. All study procedures were approved by Loughborough University’s Ethical Advisory Committee and Bradford Research Ethics Committee. Written consent was obtained from mother participants.

### Measurements

#### Demographic characteristics

Participating mothers completed a demographic (e.g., country of birth and education level) survey at baseline. Mothers’ educational levels were dichotomized into ≤ O-level (secondary education) or ≥ A-level. Mothers’ marital status (married, single, separated, divorced, or widowed) reported at the 24-month survey was dichotomized into married and other. Fathers reported self-identified ethnicity at baseline, which was used to determine fathers’ ethnicity [13]. Participants were assigned to one of the 2010 English Indices of Multiple Deprivation (IMD) quintiles, which is a national index to measure deprivation levels in small areas of England (the highest quintile is the most deprived) [20].

### Parenting practices

Adopting the existing PA and screen time parenting practice framework for preschool-aged children [21], we investigated *supportive* practices (e.g., modeling, logistic support) and *controlling* practices (e.g., limits of PA or TV viewing). To assess parenting practices, the 24-month survey included questions adopted from the validated Southampton Women’s Survey Questionnaire [22]. PA parenting practices [21] that were evaluated included implicit modeling (i.e., maternal value of PA), explicit modeling (i.e., parental modeling of PA [23], co-activity, and verbal encouragement), logistic support, and PA restrictions. TV viewing parenting practices [21] that were evaluated included implicit modeling (i.e., maternal value of limiting TV viewing), explicit modeling (i.e., parental modeling of TV viewing [24]), family culture (i.e., exposure to TV), and TV viewing restrictions. The question items and measures for each of these components are described in Table 1.

**Table 1:** Physical Activity and TV viewing parenting practice components measured

Component	Question item(s)	Variable(s)
Maternal value for PA	“I think it’s important that my child is physically active.”	Dichotomous variable: agree or disagree
Maternal modeling for PA	International PA questionnaire (IPAQ).	Weekly minutes (minutes/week)
Co-activity	“In the last month, how often have you or your partner done a physical activity or played in a physically active way with your child?”	Weekly frequency (times/week)
Verbal encouragement for PA	“In the last month, how often have you or your partner encouraged your child to play physically active games?	Weekly frequency (times/week)
Logistic support for PA	“In the last month, how often have you or your partner taken your child to places where he/she can be physically active?”	Weekly frequency (times/week)
PA restriction	“In the last month, how often was the child restricted from engaging in PA for the following reasons: (1) the cost of clubs or facilities, (2) it is difficult to travel to places where my child can be physically active, (3) the weather, (4) I am too busy, (5) I am scared that my child will get hurt, (6) there are no other children to play with, and (7) there is no adult to supervise the children whilst playing.”	Weekly frequency (times/week) for each of the seven questions
Maternal value for limiting TV viewing	“I think it’s important that my child does not watch too much TV.”	Dichotomous variable: agree or disagree
Maternal modeling for TV viewing	“Over the last month, on average how many hours per day did you watch TV or DVDs: a) on a weekday before 6 pm; b) on a weekday after 6 pm; c) on a weekend day before 6 pm; and d) on a weekend day after 6 pm?”.	Daily hours (hours/day) calculated: ([*a* + *b*] x 5 + [*c* + *d*] x 2) ÷ 7
Exposure to TV	“In the past month, has your child watched TV at mealtimes?”	Weekly frequency (times/week)
TV viewing restriction	“In the past month, how often have you or your partner limited the time your child spends watching TV/DVDs?”	Weekly frequency (times/week)

*MVPA* Moderate- and vigorous-intensity physical activity, *PA *Physical activity

### Child PA and TV viewing behaviors

Mothers reported child PA behaviors at ages 24 and 36 months in response to questions adopted from the EPIC Norfolk questionnaire [24]: weekly frequencies (times/week) of (a) walking to get from place to place (hereafter referred to as “walking”), and (b) PA that makes the child sweat or breathe harder (hereafter referred to as “intensive PA”). Mothers reported children’s TV viewing time at ages 24 and 36 using the same set of TV viewing questions for mothers (Table 1). Children’s daily TV viewing hours (hours/day) were calculated using the same equation as mother’s daily TV viewing hours presented in Table 1.

### Statistical analyses

All analyses were conducted using SAS 9.4 (Cary, NC). Descriptive analyses, including frequency and distribution analyses, were conducted. Due to small sample sizes for the highest (5th ) IMD quintile group (*n* = 5 for SAB and *n* = 43 for WB), we combined the 4th and 5th quintile groups. We conducted bivariate analyses (i.e., chi-square tests and t-tests) to compare parenting practices, child PA, and child TV viewing between the study groups. Among SAB families, we conducted t-tests to compare PA parenting practices by child sex to explore whether they differed by child sex.

Multivariable linear regression models were built to predict the frequencies of walking and intensive PA (times/week) at child ages 24 and 36 months by the study group variable (foreign-born SAB, UK-born SAB, and WB [reference group]) and the PA parenting practice variables. Another set of multivariable linear regression models were built to predict TV viewing time (hours/day) at child ages 24 and 36 months by the study group variables and the TV viewing parenting practice variables. All regression models were adjusted for child sex, mothers’ education, and IMD levels to account for potential differences by sex and socioeconomic status. A significance level was set at 0.01 (two-sided).

## Results

Of the 1,641 mothers with SAB (59.2%) or WB (40.8%) ethnicity, we excluded 492 who did not complete the 24-month survey, which resulted in 1,149 mothers (694 SAB [60.4%] and 455 WB [39.6%]) for data analysis. The 492 excluded mothers had similar distributions of ethnicity (chi-square *p* = 0.12) and IMD quintiles (chi-square *p* = 0.32) with the 1,149 included mothers. Of the 1,149 mothers, 97 (8.4%) did not complete the 36-month survey.

Among the 694 SAB mothers, 418 (60.2%) were foreign-born and 276 (39.8%) were UK-born. In terms of fathers’ ethnicity, 691 (99.6%) fathers in the SAB groups were SA and 435 (95.6%) fathers in the WB group were White. Table 2 presents demographic characteristics of the study participants. Mothers’ average age was similar across the three study groups: 30.6 years (range: 20–51 years) in foreign-born SAB; 29.2 years (19–45 years) in foreign-born SAB; and 29.5 years (17–46 years) in WB. Among 694 SAB mothers, 613 (88.3%) identified as being of Pakistani origin, 50 (7.2%) Indian origin, 25 (3.6%) Bangladeshi origin, and 6 (0.9%) with Bhutanese, Nepali, or Sri Lankan origins.

**Table 2:** Comparisons of characteristics among three study groups

	Foreign-born South Asian British (*n* = 418)	UK-born South Asian British (*n* = 276)	White British (*n* = 455)
	n (%)	n (%)	n (%)
**Mother’s marital status**			
Married	408 (97.6)	257 (93.1)	204 (44.8)
Single, separate, or divorced	10 (2.4)	19 (6.9)	251 (55.2)
**Mother’s education**			
O-level or lower (≤ secondary education)	278 (66.5)	153 (55.4)	289 (63.5)
A-level or higher (> secondary education)	140 (33.5)	123 (44.6)	166 (36.5)
**IMD 2010 quintile**			
1st quintile (least deprived)	322 (77.0)	222 (80.4)	232 (51.0)
2nd quintile	71 (17.0)	32 (11.6)	107 (23.5)
3rd quintile	21 (5.0)	21 (7.6)	73 (16.0)
4th or 5th quintile (most deprived)	4 (1.0)	1 (0.4)	43 (9.5)
**Child sex**			
Male	201 (48.1)	143 (51.8)	218 (47.9)
Female	217 (51.9)	133 (48.2)	237 (52.1)

*IMD* Indices of multiple deprivation

The foreign-born SAB group showed the lowest frequencies of PA verbal encouragement (3.7 ± 3.1 times/week) and logistic support (1.5 ± 1.8 times/week), followed by the UK-born SAB group (4.4 ± 2.7 and 2.0 ± 1.8 times/week, respectively) and the WB group (5.2 ± 2.4 and 3.0 ± 2.0 times/week, respectively) (*p* < 0.01; Table 3). PA restriction was most frequent in the foreign-born SAB group (7.8 ± 7.7 times/week), followed by the UK-born SAB group (4.7 ± 5.8 times/week) and the WB group (2.7 ± 4.4 times/week) (*p* < 0.01). Foreign-born and UK-born SAB mothers reported that their children less frequently walked to get from place to place at ages 24 and 36 months, compared to children of WB mothers (*p* < 0.01). PA parenting practices and child engagement in walking did not differ by child sex within the SAB groups (Supplementary Table 1).

**Table 3:** Comparison of physical activity parenting practices and child physical activity among three study groups

	Foreign-born South Asian British (*n* = 418)	UK-born South Asian British (*n* = 276)	White British (*n* = 455)
	n (%) or Mean ± SD		
**Supportive parenting practices**			
Maternal value for child PA	393 (94.0)	266 (96.4)	443 (97.4)
Mother MVPA, minutes/week	127 ± 198^b^	151 ± 212^c^	276 ± 297
Co-activity, times/week	4.3 ± 2.9^b^	4.4 ± 2.6^c^	5.4 ± 2.1
Verbal encouragement, times/week	3.7 ± 3.1^a,b^	4.4 ± 2.7^c^	5.2 ± 2.4
Logistic support, times/week	1.5 ± 1.8^a,b^	2.0 ± 1.8^c^	3.0 ± 2.0
**Controlling parenting practices: PA Restriction due to, times/week**			
1. cost of clubs or facilities	0.3 ± 1.1	0.3 ± 1.1	0.4 ± 1.2
2. difficult to travel to places where my child can be physically active	1.1 ± 2.1^a,b^	0.6 ± 1.6	0.4 ± 1.3
3. the weather	2.3 ± 2.3^a,b^	1.4 ± 1.8	1.1 ± 1.7
4. too busy	1.5 ± 2.4^a,b^	0.9 ± 1.8^c^	0.4 ± 1.0
5. scared that my child will get hurt	1.6 ± 2.6^a,b^	0.5 ± 1.5^c^	0.1 ± 0.8
6. no other children to play with	0.5 ± 1.4^b^	0.4 ± 1.3^c^	0.2 ± 0.8
7. no adult to supervise the child whilst playing	0.5 ± 1.6^b^	0.4 ± 1.5^c^	0.1 ± 0.8
Sum of PA restriction frequencies	7.8 ± 7.7^a,b^	4.7 ± 5.8^c^	2.7 ± 4.4
**Child PA, days/week**			
Walking at age 24 months	3.5 ± 2.7^b^	3.9 ± 2.6^c^	4.8 ± 2.4
Walking at age 36 months^¥^	4.1 ± 2.5^a,b^	4.7 ± 2.6^c^	5.8 ± 2.0
Intensive PA at age 24 months	0.6 ± 1.7^a,b^	1.3 ± 2.5	1.7 ± 2.7
Intensive PA at age 36 months^¥^	0.7 ± 2.0^b^	1.2 ± 2.3^c^	2.1 ± 2.9

^a^*p*<0.01 between the foreign-born and UK-born SAB groups, ^b^*p*<0.01 between the foreign-born SAB and White British groups, ^c^*p*<0.01 between the UK-born SAB and White British groups; ^¥^missing *n* = 97; *MVPA *Moderate- and vigorous-intensity physical activity, *PA *Physical activity, *SD *Standard deviation

The foreign-born SAB group most frequently allowed their children to watch TV at mealtimes (4.0 ± 3.1 times/week), followed by the UK-born SAB group (2.6 ± 2.8 times/week) and the WB group (2.1 ± 2.6 times/week) (*p* < 0.01; Table 4). Children of the foreign-born SAB mothers tended to engage in most TV viewing time at ages 24 and 36 months, followed by children of UK-born SAB and WB mothers.

**Table 4:** Comparison of TV viewing parenting practices and child TV viewing among three study groups

	Foreign-born South Asian British (*n* = 418)	UK-born South Asian British (*n *= 276)	White British (*n* = 455)
**Supportive parenting practices**	n (%) or Mean ± SD		
Maternal value for limiting TV viewing	316 (75.6)	207 (75.0)	358 (78.7)
Maternal TV viewing, hours/day	2.4 ± 1.6^b^	2.4 ± 1.6^c^	2.7 ± 1.6
Exposure to TV during a mealtime, times/week	4.0 ± 3.1^a,b^	2.6 ± 2.8^c^	2.1 ± 2.6
**Controlling parenting practices**			
Limit child watching TV, times/week	2.1 ± 2.8^b^	2.6 ± 2.9^c^	3.4 ± 3.1
**Child TV viewing, hours/day**			
TV viewing at age 24 months	2.0 ± 1.5^b^	1.7 ± 1.4	1.5 ± 1.2
TV viewing at age 36 months^¥^	2.5 ± 1.7^a,b^	2.1 ± 1.5^c^	1.7 ± 1.1

^a^*p*<0.01 between the foreign-born and UK-born SAB groups, ^b^*p*<0.01 between the foreign-born SAB and White British groups, ^c^*p*<0.01 between the UK-born SAB and White British groups; ^¥^missing *n* = 97, *SD *Standard deviation

The multivariable linear regression models for the child PA outcomes showed that children of foreign-born SAB mothers engaged in walking 0.9 times/week less at age 24 months and 1.4 times/week less at age 36 months, compared to their WB counterparts (*p* < 0.01; Table 5). The frequency of intensive PA was also significantly lower among children of foreign-born SAB mothers (Supplementary Table 2).

**Table 5:** Multivariable linear regression models for the frequency of walking to get from place to place at child ages 24 and 36 months

	Walking at 24 months, days/week (*n* = 1,149)		Walking at 36 months, days/week (*n* = 1,052)	
	Estimate ± SE	*p*-value	Estimate ± SE	*p*-value
Intercept	3.9 ± 0.4	< 0.01	5.0 ± 0.4	< 0.01
Child sex: male vs. female	0.2 ± 0.1	0.12	-0.05 ± 0.1	0.76
Maternal education: ≥A-level vs. ≤O-level	-0.2 ± 0.2	0.18	0.01 ± 0.2	0.96
IMD: 2nd vs. 1st quintile	0.2 ± 0.2	0.39	-0.1 ± 0.2	0.55
3rd vs. 1st quintile	-0.2 ± 0.3	0.44	-0.4 ± 0.3	0.12
4-5th vs. 1st quintile	-0.2 ± 0.4	0.69	0.3 ± 0.4	0.40
Maternal ethnicity: Foreign-born SAB vs. WB	-0.9 ± 0.2	< 0.01	-1.4 ± 0.2	< 0.01
Maternal ethnicity: UK-born SAB vs. WB	-0.7 ± 0.2	< 0.01	-0.9 ± 0.2	< 0.01
Maternal value for PA	-0.1 ± 0.4	0.78	0.1 ± 0.4	0.80
Maternal MVPA, hours/week	0.04 ± 0.02	0.03	0.01 ± 0.02	0.59
Co-activity, times/week	-0.03 ± 0.04	0.43	-0.01 ± 0.03	0.88
Verbal encouragement, times/week	0.1 ± 0.03	< 0.01	0.1 ± 0.03	0.01
Logistic support, times/week	0.2 ± 0.01	< 0.01	0.1 ± 0.04	< 0.01
PA restriction, times/week	-0.01 ± 0.01	0.48	-0.01 ± 0.01	0.44

*IMD *Index of multiple deprivation, *MVPA *Moderate- and vigorous-intensity physical activity, *PA *Physical activity, *SAB *South Asian British, *SE *Standard error, *WB *White British

The multivariable linear regression models for the child TV viewing outcome showed that one additional exposure to TV during a mealtime per day was associated with 55 min/day higher TV viewing time at age 24 months (*p* < 0.01; Table 6). Children of foreign-born SAB mothers had significantly higher TV viewing time, compared to their WB counterparts: 21 min/day higher at age 24 months and 42 min/day higher at age 36 months.

**Table 6:** Multivariable linear regression models for TV viewing at child ages 24 and 36 months

	TV viewing at 24 months, hours/day (*n* = 1,149)		TV viewing at 36 months, hours/day (*n* = 1,052)	
	Estimate ± SE	*p*-value	Estimate ± SE	*p*-value
Intercept	0.9 ± 0.1	< 0.01	1.3 ± 0.2	< 0.01
Child sex: male vs. female	-0.03 ± 0.1	0.68	-0.1 ± 0.1	0.31
Maternal education: ≥A-level vs. ≤O-level	-0.02 ± 0.1	0.77	-0.1 ± 0.1	0.13
IMD: 2nd vs. 1st quintile	-0.1 ± 0.1	0.57	-0.1 ± 0.1	0.52
3rd vs. 1st quintile	-0.01 ± 0.1	0.93	-0.3 ± 0.1	0.08
4-5th vs. 1st quintile	-0.05 ± 0.2	0.81	-0.1 ± 0.2	0.74
Maternal ethnicity: Foreign-born SAB vs. WB	0.3 ± 0.1	< 0.01	0.7 ± 0.1	< 0.01
Maternal ethnicity: UK-born SAB vs. WB	0.2 ± 0.1	0.02	0.5 ± 0.1	< 0.01
Maternal value for limiting TV viewing	-0.4 ± 0.1	< 0.01	-0.1 ± 0.1	0.19
Maternal TV viewing, hours/day	0.2 ± 0.02	< 0.01	0.2 ± 0.03	< 0.01
Exposure to TV during a mealtime, times/day	0.9 ± 0.1	< 0.01	0.7 ± 0.1	< 0.01
TV viewing restriction, times/day	-0.2 ± 0.1	0.01	0.04 ± 0.1	0.69

*IMD *Index of multiple deprivation, *SAB *South Asian British, *SE *Standard error, *WB *White British

## Discussion

In an examination of PA parenting practices for young children among a sample of mother-child dyads in the UK, we found that foreign-born SAB parents least frequently encouraged their children to engage in PA. At the same time, foreign-born SAB parents most frequently restricted their children from being physically active, partly because of concerns related to weather, injury, and transportation. Less frequent PA-supportive parenting practices was associated with less frequent walking among children. Even after accounting for socioeconomic status and PA parenting practices, children of foreign-born SAB mothers engaged in walking less frequently, compared to their WB counterparts. In an examination of TV viewing parenting practices for young children, exposure to TV at mealtimes was most frequent among children of foreign-born SAB mothers. After accounting for socioeconomic status and TV viewing parenting practices, children of foreign-born SAB mothers had higher TV viewing time, compared to their WB counterparts.

This is one of the first studies to report on PA and sedentary behavior-related parenting practices among SAB parents of toddlers. The strength of this study includes the use of a large study sample obtained from a population-based cohort study. The large sample size enabled us to examine differences between the foreign-born and UK-born SAB groups as well as between the UK-born SAB and BW groups. A longitudinal examination of child PA and TV viewing at age 24 and 36 months allowed us to provide evidence that differences in PA and TV viewing behaviors between SAB and WB children could become larger as children grew older -- from age 24 to 36 months.

Our findings of less supportive and more restrictive PA-related parenting practices for young children among SAB families are consistent with prior findings for preschool and school-aged SAB children [14, 15]. Specifically, this study revealed that transportation challenges and weather and injury concerns (i.e., “scared that my child gets hurt”) were common reasons for restrictive PA parenting practices among foreign-born SAB parents. On the other hand, we found that UK-born SAB parents were more supportive and less restrictive of child PA, compared with foreign-born SAB parents. Consistent with a prior study [13], the preset study support that both SA ethnicity and acculturation play a role in PA-related parenting practices. A qualitative study of SAB parents [15] revealed that because PA has a low level of importance in the SA community, PA is not encouraged from young age in SAB families. SAB parents may become more supportive of child PA and less concerned about potential barriers such as weather and injury as they become more acculturated. Second-generation SAB adults may adopt more positive attitudes and practices related to PA through the process of acculturation and education within UK schools, and as a result adopt norms similar to WB adults [7]. Neighborhood factors and childcare environment, which were not accounted for in the present study, might partly explain the differences in parenting practices and children’s PA between SAB and WB [7].

The literature recognizes SA’s gender norms related to engagement in PA, such that PA is generally less emphasized for women and girls compared to men and boys [9]. We found no difference in PA parenting practices by child sex for neither foreign nor UK-born SAB parents, which may in part be due to the children’s young age. Gender norms may become more relevant as girls become older [14]. These data are not able to further evaluate whether and how gender norms are differentially reflected in parenting practices by child sex among SAB families. Future research should investigate how and in which childhood stage gender norms are reflected in parenting practices among SAB families and whether and how those change with acculturation.

This study identified that children of foreign-born SAB mothers less frequently engaged in walking to/from places, compared to WB children, at both 24 and 36 months of age. Even after accounting for the various parenting practices measured in this study, a mother’s SA ethnicity (both foreign-born and US-born) remained a significant factor associated with her child’s lower participation in walking. Considered together with the previous finding of a lower likelihood of walking to/from school among SAB school-aged children [14, 25], these results suggest that low engagement in walking for transportation among SAB children could represent a pattern that begins in early childhood and continues over time into later childhood.

We also found that children of foreign-born SAB mothers rarely engaged in intensive PA. It has been previously reported that both children and adults of SA ethnicity engage in very little vigorous PA [26, 27] and less frequently engage in organized sports, compared to white children and adults [28]. Our finding of higher intensive PA among children of UK-born SAB mothers than children of foreign-born SAB mothers is consistent with a previous study [28] which reported that SA American parent acculturation was positively associated with children’s sports participation, an association related to the parent’s value placed on sports achievement.

The literature suggests that exposure to TV during a mealtime is a strong predictor of high TV viewing time among young children [29]. This study revealed that more frequent exposure to TV at mealtimes occurred among SAB children than WB children, which is consistent with a prior report [13]. SAB parents also reported less frequently limiting their children’s TV viewing time. In conjunction with these practices, children of foreign-born SAB mothers watched TV more than WB children: 30 min/day more at age 24 months and 50 min/day more at age 36 months. Although foreign-born SAB mothers reported watching slightly less TV than did WB mothers, they less frequently limited their young children’s TV viewing. Allowing children to watch more TV may partly be explained by the desire for children to learn language and British culture and cues by watching TV. Indeed, quality TV programming could provide an additional route to early language and literacy skills for young children [30, 31]. Despite those potential benefits, excessive exposure to TV in early childhood is known to have harmful effects on development and health [32–35]. Educating SA families about the importance of limiting TV viewing via screen time rules for young children (i.e., no screen time at mealtimes) could help SA parents better support their children in establishing healthier sedentary behavior habits starting in early childhood.

Several limitations of this study should be acknowledged. First, child PA and TV viewing behaviors were not objectively measured, but proxy-reported by their mothers, which is prone to measurement error and social desirability and recall biases. Second, this study did not account for all parenting practice components that are suggested to influence PA and sedentary behaviors [21]; therefore, residual confounding might have affected the association observed between SA ethnicity and child PA and TV viewing. Third, because this cross-sectional observation is unable to provide evidence of temporal relationships between children’s PA and TV viewing behaviors and parenting practices, it does not rule out a potential reverse causation: for example, in response to a child’s temperament (e.g., high activity), parents might have changed their parenting practices (e.g., taking the child to places where the child can be physically active). Fourth, the present study did not examine other screen time behaviors, such as time spent on mobile devices. Further, this study used data collected over a decade ago. It is possible that parenting practices might have changed over time. There are likely additional considerations now with the ubiquitous presence of mobile devices. Nonetheless, our findings remain relevant to current screen media use among young children, as TV viewing still makes up over three quarters of total screen time among young children [36] and children’s TV viewing and other media use have common correlates with parental factors (e.g., parental modeling and parental attitudes). Fifth, we did not consider the ethnicity of fathers and partners in defining study groups. However, only a small proportion of the fathers had a different ethnic background from the mothers: 0.4% in the SAB groups and 4.4% in the BW group. Lastly, the SAB sample mostly included Pakistanis. As there may be similarities and differences between various countries that comprise the SA diaspora, caution is required to generalize the results to other SA subgroups.

## Conclusions

This study demonstrated that SAB parents, particularly those that are foreign-born, apply parenting practices for their young children that are less supportive of PA and more supportive of TV viewing, and their children have lower PA and higher TV viewing time, compared with their WB counterparts.

### Supplementary Information

**Additional file 1: Supplementary Table 1.** Comparison of parenting practices and child physical activity by child sex among South Asian mothers and their children. **Supplementary Table 2.** Multivariable linear regression models for the frequency of intensive physical activity at ages 24 and 36 months.

## Data Availability

The datasets used and/or analysed during the current study available from the corresponding author on reasonable request.

## Abbreviations

WB: White British
IMD: Indices of multiple deprivation
MVPA: Moderate- and vigorous-intensity physical activity
PA: Physical activity
SA: South Asian
