# Supplementary material for: Physical activity and TV viewing parenting practices for toddlers among South Asian and white families in the UK: born in Bradford 1000 study
Source: BMC Public Health. 2023 Aug 22;23:1590. doi: 10.1186/s12889-023-16522-w (PMC10463369; doi:10.1186/s12889-023-16522-w)
Supplement: Supplementary file 1 — Additional file 1: Supplementary Table 1. Comparison of parenting practices and child physical activity by child sex among South Asian mothers and their children. Supplementary Table 2. Multivariable linear regression models for the frequency of intensive physical activity at ages 24 and 36 months. [file 12889_2023_16522_MOESM1_ESM.docx]

**Supplementary Data**

Supplementary Table 1. Comparison of parenting practices and child physical activity by child sex among South Asian mothers and their children.

|  | Male (n=344) | Female (n=350) | P-value |
| --- | --- | --- | --- |
|  | n (%) or Mean±SD | |  |
| **Supportive parenting practices** |  |  |  |
| Maternal value for child PA | 323 (93.9) | 336 (96.0) | 0.21 |
| **Parenting practices, times/week** |  |  |  |
| Co-activity, times/week | 4.4±2.7 | 4.3±2.8 | 0.71 |
| Verbal encouragement, times/week | 4.1±2.9 | 3.9±2.9 | 0.52 |
| Logistic support, times/week | 1.8±1.8 | 1.6±1.7 | 0.13 |
| **Controlling parenting practices: PA Restriction due to, times/week** |  |  |  |
| 1. cost of clubs or facilities | 0.3±0.9 | 0.3±1.2 | 0.50 |
| 2. difficult to travel to places where my child can be physically active | 0.9±1.9 | 1.0±.2.1 | 0.51 |
| 3. the weather | 1.8±2.1 | 2.0±2.1 | 0.35 |
| 4. too busy | 1.3±2.1 | 1.3±2.2 | 0.71 |
| 5. scared that my child will get hurt | 1.1±2.3 | 1.2±2.4 | 0.48 |
| 6. no other children to play with | 0.5±1.5 | 0.4±1.2 | 0.16 |
| 7. no adult to supervise the child whilst playing | 0.5±1.5 | 0.5±1.5 | 0.98 |
| **Child PA, days/week** |  |  |  |
| Walking at age 24 months | 3.8±2.7 | 3.5±2.6 | 0.23 |
| Walking at age 36 months | 4.3±2.5 | 4.3±2.6 | 0.88 |
| Intensive PA at age 24 months | 1.2±2.3 | 0.6±1.8 | <0.01 |
| Intensive PA at age 36 months | 1.1±2.4 | 0.7±1.9 | 0.01 |

PA, physical activity; SD, standard deviation.

Supplementary Table 2. Multivariable linear regression models for the frequency of intensive physical activity at ages 24 and 36 months.

|  | Intensive PA at 24 months, days/week  (n=1,149) | | Intensive PA at 36 months, days/week  (n=1,052) | |
| --- | --- | --- | --- | --- |
|  | Estimate±SE | p-value | Estimate±SE | p-value |
| Maternal ethnicity: Foreign-born SAB vs. WB | -0.9±0.2 | <0.01 | -0.9±0.2 | <0.01 |
| Maternal ethnicity: UK-born SAB vs. WB | -0.2±0.2 | 0.20 | -0.6±0.2 | <0.01 |
| Maternal value for PA | -0.03±0.3 | 0.93 | -0.3±0.4 | 0.45 |
| Maternal MVPA, hours/week | 0.01±0.02 | 0.39 | 0.03±0.02 | 0.10 |
| Co-activity, times/week | -0.001±0.03 | 0.97 | 0.03±0.04 | 0.34 |
| Verbal encouragement, times/week | 0.05±0.03 | 0.11 | 0.05±0.03 | 0.14 |
| Logistic support, times/week | 0.03±0.04 | 0.41 | 0.1±0.04 | <0.01 |
| PA restriction, times/week | -0.003±0.01 | 0.76 | -0.01±0.01 | 0.29 |

All estimates were adjusted for sex, maternal education, and IMD 2010 quintile.

Intensive PA was defined physical activity that makes the child sweat or breath harder.

IMD, index of multiple deprivation; MVPA, moderate- and vigorous-intensity physical activity; PA, physical activity; SAB, South Asian British; SE, standard error; WB, White British.
